# Public health round-up

**DOI:** 10.2471/BLT.21.010721

**Published:** 2021-07-01

**Malaria diagnostic threatened**

A UNICEF-trained community health agent shows the rapid diagnostic test he used to check for malaria parasites in a 10-month-old baby in the village of Bidjir, Chad. Rapid diagnostic tests have transformed malaria control, enabling better targeting of treatment and improved surveillance. A widely used test is now likely to become ineffective because of a genetic mutation in *Plasmodium falciparum* parasites.

**Figure:**
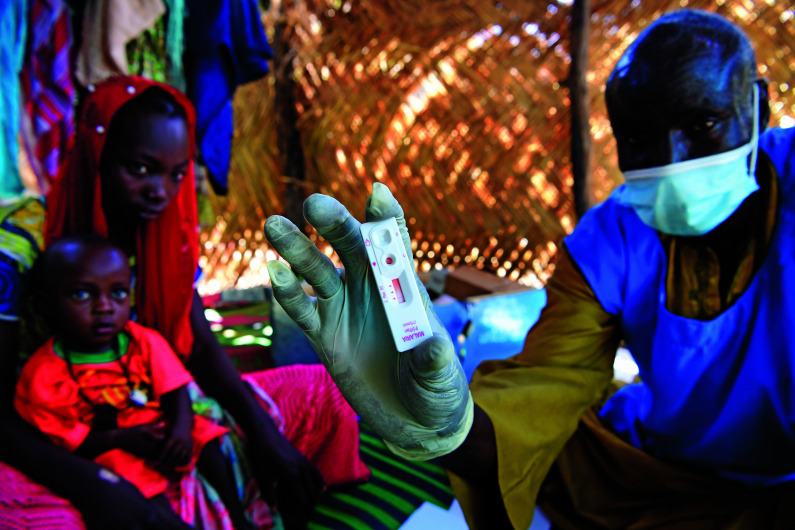
UNICEF/Frank Dejongh

## G7 COVID-19 vaccine commitment

Group of Seven (G7) leaders ended their annual summit with a commitment to donate 870 million coronavirus disease 2019 (COVID-19) vaccine doses, to be delivered over the next twelve months.

Most of the doses will be delivered through the COVAX facility, the vaccine pillar of the Access to COVID-19 Tools (ACT) Accelerator, a global collaboration to accelerate the development, production and equitable access to new COVID-19 diagnostics, therapeutics and vaccines.

On 13 June, the leaders reaffirmed their support for all pillars of the ACT-Accelerator while also indicating their intention to work with the private sector, the G20 and other countries to increase their vaccine contributions over the months to come. Since their Early Leaders’ Summit in February 2021, the G7 has committed to donate one billion doses in total.

Speaking to leaders at the meeting, WHO Director-General Tedros Adhanom Ghebreyesus said “We welcome the generous announcements about donations of vaccines and thank leaders. But we need more, and we need them faster.”

Increased support and investment are also required for the ACT-Accelerator, total funding for which is currently US$ 15.1 billion, some US$16 billion short of the amount required to fully fund its work this year.

https://bit.ly/3iDAmRC

## Malaria diagnostic challenge

A crucial malaria rapid diagnostic test is under serious threat due to the emergence of *Plasmodium falciparum* parasites not expressing the protein that the test targets.

Rapid diagnostic tests have transformed malaria control in recent years, enabling better targeting of treatment and improved surveillance. Globally, 2.7 billion of the tests were sold between 2010 and 2019, the majority distributed in sub-Saharan Africa to diagnose *P. falciparum* infection by targeting one of its antigens, histidine-rich protein 2 (HRP2).

According to the Malaria Policy Advisory Group, the increased prevalence of HRP2 gene deletions in *P. falciparum* in all endemic countries risks rendering HRP2-based tests ineffective.

On 27 May, the group called for urgent action to address the problem, emphasizing the need for countries to start and maintain surveillance and where appropriate change to quality-assured non-HRP2 diagnostic tests to prevent disease and deaths.

https://bit.ly/2TVeiaM

## Sinovac-CoronaVac approved for emergency use

WHO validated the Sinovac-CoronaVac COVID-19 vaccine for emergency use on 1 June, giving countries, funders, procuring agencies and communities the assurance that it meets international standards for safety, efficacy and manufacturing quality.

Containing inactivated severe acute respiratory syndrome coronavirus 2 (SARS-CoV-2), the two-dose vaccine can be transported and stored at 2–8 °C which makes it suitable for distribution in countries lacking ultra-cold-chain capacity.

The vaccine is produced by the Beijing-based pharmaceutical company Sinovac.

https://bit.ly/3cCTRG6

## Strategy to end polio

The Global Polio Eradication Initiative launched a new polio eradication strategy that underscores the need to get eradication efforts back on track. Launched 10 June, the strategy calls for further integration of polio activities with essential health services, and building closer partnerships with high-risk communities to co-design immunization events and better meet their health needs. The strategy also proposes applying a gender equality lens to programme implementation, recognizing the important contribution made by female workers, notably in building community trust and improving vaccine acceptance.

While polio cases have fallen 99.9% since 1988, polio remains a Public Health Emergency of International Concern. Persistent barriers to reaching every child with polio vaccines and the pandemic have contributed to an increase in wild polio cases, while the increase in vaccine-derived polio cases is a growing concern. Last year, 1226 cases were recorded compared to 138 in 2018.

https://bit.ly/3wsXYN6

## Call to fund pandemic response and economic recovery

The International Monetary Fund, World Bank, WHO and World Trade Organization called for urgent action from government leaders to finance a new US$ 50 billion roadmap to accelerate the equitable distribution of health tools needed to help end the pandemic while laying the foundations for a global economic recovery.

In an appeal published on 1 June, the leaders of the agencies said governments need to act without further delay or risk continued waves and explosive outbreaks of COVID-19, as well as the emergence of more transmissible and deadly virus variants which could undermine the global recovery.

“By now it has become abundantly clear there will be no broad-based recovery without an end to the health crisis,” the leaders said, stressing that ensuring global access to vaccination is key.

https://bit.ly/3cEXdIy

## Explaining vaccine regulation

The International Coalition of Medicines Regulatory Authorities and WHO issued a statement for the attention of health-care professionals regarding the role of regulators in the oversight of COVID-19 vaccines. The statement describes how vaccines undergo robust scientific evaluation to determine their safety, efficacy and quality and how safety is closely and continually monitored after approval.

The global impact of the COVID-19 pandemic has resulted in an unprecedented level of public interest in and commentary on vaccine safety efficacy and regulation. Much of this commentary has taken place through mass and social media, leading at times to the dissemination of misinformation.

The 11 June statement was made to counter such misinformation and was directed towards health-care professionals in recognition of the fact that they play a central role in discussing COVID-19 vaccination with their patients.

https://bit.ly/3zrQxr0

## One Health High-Level Expert Panel formed

A new One Health High-Level Expert Panel was launched on 20 May to improve understanding of how diseases with the potential to trigger pandemics emerge and spread. Comprising 26 international experts with a range of technical knowledge, skills and experience, the panel will advise WHO, the Food and Agriculture Organization of the United Nations (UN), the World Organisation for Animal Health and the UN Environment Programme applying the One Health approach, which recognizes the links between the health of people, animals and the environment and highlights the need for a multisectoral effort to address the challenges faced.

The panel will guide the development of a new research agenda and draw up evidence-based recommendations.

https://bit.ly/2TsTl6S

## Work and stroke

Long working hours led to 745 000 deaths from stroke and ischaemic heart disease in 2016, a 29% increase since 2000, according to the latest estimates published by WHO and the International Labour Organization.

Published 17 May, the first global analysis of the loss of life and health associated with working long hours reveals that in 2016, an estimated 398 000 people died from stroke and 347 000 from heart disease as a result of having worked at least 55 hours a week.

https://bit.ly/3gm1i6V

**Cover photo**

People leaving their homes after the eruption of the Nyiragongo volcano in the city of Goma, North Kivu Province, Democratic Republic of the Congo.

**Figure:**
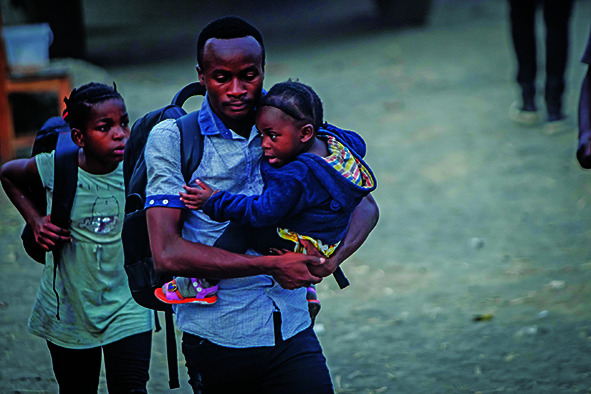
UNICEF/Guershom Ndebo

## Guidance on genetically modified mosquitoes

New WHO guidance sets out essential standards for future research and development on genetically modified mosquitoes, including standards relating to ethics, safety, affordability and effectiveness.

Launched 19 May, the guidance describes best practices to ensure that the study and evaluation of genetically modified mosquitoes as public health tools are safe, ethical and rigorous.

Malaria and other vector-borne diseases, including dengue and Zika, affect millions globally, with more than 400 000 people dying each year from malaria alone. If proven safe, effective and affordable, genetically modified vector mosquitoes could be a valuable new tool to fight these diseases.

https://bit.ly/3cEhQ86

## New global energy access report

Close to 3 billion people still have no access to clean cooking solutions, most of those people living in Asia and sub-Saharan Africa. This is according to a new energy progress report released on 7 June by WHO in collaboration with international energy agencies, the UN Department of Economic and Social Affairs and the World Bank.

The report assesses the progress made by each country on energy access, energy efficiency, renewable energy and international cooperation, providing a snapshot of how countries are progressing towards Sustainable Development Goal 7 which targets ensuring access to affordable, reliable, sustainable and modern energy for all.

https://bit.ly/3zt3TDM

**Looking ahead**

06–15 July. High-level political forum 2021. https://bit.ly/3izLVcI

12 July. Codex Alimentarius Commission meeting on Residues of Veterinary Drugs in Foods. https://bit.ly/3vgfQJk

18–21 July. 11th International AIDS Society Conference on HIV Science. https://bit.ly/3pIuN5W

